# A Bioengineered Skin Organoid Platform for Modeling Human Skin Physiology and Cytotoxicity

**DOI:** 10.21203/rs.3.rs-7989400/v1

**Published:** 2025-11-25

**Authors:** Anastasiya Gorkun-Roeder, Naresh Mahajan, Gemma Nomdedeu-Sancho, Adam Jorgensen, Kelsey Willson, Mingsong Wu, Dongdong Ji, Shay Soker, Anthony Atala

**Affiliations:** Wake Forest Institute for Regenerative Medicine; Wake Forest Institute for Regenerative Medicine; Wake Forest Institute for Regenerative Medicine; Wake Forest Institute for Regenerative Medicine; Wake Forest Institute for Regenerative Medicine; Wake Forest Institute for Regenerative Medicine; Wake Forest Institute for Regenerative Medicine; Wake Forest Institute for Regenerative Medicine; Wake Forest Institute for Regenerative Medicine

**Keywords:** skin development, chemical irritation, ultraviolet B radiation, high-throughput testing, body-on-a-chip

## Abstract

Three-dimensional in vitro skin models are developed for studying skin physiology and drug toxicology. Most currently existing models are constructed through self-aggregation or layer-by-layer assembly of fibroblasts and keratinocytes only disregarding other important skin cell types.

In this study, we used organoid fabrication technology to enable self-organization of multiple skin cell types into complex, tissue-like structures. The human skin organoids (SO) model was generated through aggregation of six primary human skin cell types—keratinocytes, melanocytes, dermal papilla cells, endothelial cells, fibroblasts, and adipocytes—under non-adhesive, gravity-driven culture conditions. The SOs exhibited spontaneous morphogenesis and developed compartmentalized structures resembling the native skin, including a stratified epidermis and a dermal–hypodermal core.

The SOs maintained their skin-like organization for at least 21 days in culture and demonstrated key functional properties, including epidermal barrier integrity), active retinol metabolism, and responsiveness to chemical toxicity and ultraviolet radiation. In addition, SOs supported melanogenesis and vasculogenesis.

In conclusion, we developed a self-organizing, multi cell type skin organoid system that recapitulates essential features of human skin architecture and function in vitro. Its complexity, scalability, and physiological relevance makes it a valuable platform for studies in skin physiology, toxicology, regenerative medicine, and disease modeling, including fibrotic skin disorders and skin cancer.

## 1 Introduction

The skin is the first line of defense against external threats, and it has diverse functions such as thermoregulation, and tactile and thermal sensations. Reproducing the skin’s intricate anatomy and complex tissue physiology *in vitro* has been extremely challenging, as models must be able to recapitulate specific skin functional features such as barrier function, pigmentation, vascularization, and absorption (Quantin et al., 2022; Portugal-Cohen et al., 2023; Rimal et al., 2024). The epidermal barrier is regarded as the basic function of the skin. It acts as a protective shield, preventing harmful substances from entering the body, retaining moisture (Baker et al., 2023), and regulating body temperature. Skin pigmentation represents the next layer of protection. Melanocytes residing in the basal layer of the epidermis produce the ultraviolet radiation (UVR) protective pigment melanin, which is delivered to up to 40 surrounding keratinocytes through the extension of dendrites (Lin, Fisher 2007; Bento-Lopes et al., 2023). Mimicking this process of melanogenesis *in vitro* has been one of the main hurdles in developing pigmentation of in vitro skin models s (Zurina et al., 2020; Gendreau et al., 2013).

Studying how toxic, and pharmacological agents affect skin homeostasis is crucial to tailoring drug development or finding counteractive measures. In addition, UVR is one of the most significant yet invisible environmental hazards to skin health, directly causing damage to different cellular macromolecules, including DNA, and indirectly generates reactive oxygen species (ROS), disrupting signaling cascades which ultimately lead to apoptosis. (Tanveer et al., 2023). Due to the physiological constraints and ethical considerations linked to conventional animal testing techniques, there has been increased interest in the development of alternative skin models. *In vitro* human skin models offer a more economic and precise depiction of human skin, enabling researchers to examine the impacts of different drugs and treatments without causing harm to animals or relying exclusively on human volunteers (Portugal-Cohen et al., 2023, Morales et al., 2016). The main requirements for validation of *in vitro* skin irritation models are summarized in OECD guidelines, *Test No. 439: In Vitro Skin Irritation: Reconstructed Human Epidermis Test Method* (OECD, 2021) that can be used as a reference as no standards have been developed for spherical skin organoid models.

Despite the wide availability of skin models in the market, (Footner et al., 2023), including pigmented skin equivalents (Gledhill et al., 2015; Branquinho et al., 2020; Zoio et al., 2021; Goncalves et al., 2022; Cohen et al., 2023; Goncalves et al., 2023), and epidermal barrier models for the assessment of acute skin irritation (Portes et al., 2022), there are no standardized approaches for modeling skin photodamage *in vitro*. In addition to mechanical and pigment protection, the skin provides controlled absorption, metabolism, and distribution of active substances (McKnight et al., 2022). The current regulations for *in vitro* skin absorption analysis involve assessing the diffusion of substances into and over the skin using either non-viable skin to test diffusion alone or fresh, metabolically active skin to evaluate both diffusion and skin metabolism. These indications, which are outlined in the OECD guidelines for the testing of chemicals No.428 “Skin absorption: *in vitro* method”, 2004 (OECD, 2004), should also be considered when generating in vitro human skin models.

Despite the advances made in the field, the main disadvantage of the conventional *in vitro* skin models is the inability to replicate all four specified skin functions (barrier function, pigmentation, vascularization, and absorption) simultaneously in one construct. Full-thickness skin equivalents have limited oxygen and nutrition, which only allows the inclusion of a restricted range of cell types. Besides being time and labor-consuming, the layer-by-layer biofabrication approach used to build these constructs excludes the natural cell-cell interactions that drive native morphogenesis (Footner et al., 2023).

Organoid technologies are high-throughput and mostly rely on self-assembly to establish tissue-specific boundaries. To date, SOs have been generated from adult skin stem cells or pluripotent stem cells; however, these models were specifically designed to produce skin derivatives such as hair follicles (Lee et al., 2020; Su et al., 2019; Lei et al., 2023) or sweat glands (Diao et al., 2019) and did not replicate native skin anatomical organization and functionality. Therefore, current *in vitro* SOs cannot fulfill the demand for relevant alternatives to animal testing. Here, we report the generation of an SO model using primary cells from the three skin layers capable of self-organizing and recapitulating native skin architecture and functionality. The model can respond to chemical and UVR insults, emphasizing its potential to serve as a tool for drug, chemical, and cosmetic testing.

## 2 Methods And Materials

### Human primary cell isolation

Keratinocytes, melanocytes, fibroblasts, and pre-adipocytes were isolated from discarded human foreskin (Cell Culture Systems from Discarded Operating Room Tissue, IRB00007586). To avoid individual differences between samples cells were collected from over 10 random patients under 1 year old and pooled together for the further experiments. All skin samples underwent the same cell isolation procedure. The skin was washed three times in Dulbecco’s Modified Eagle Medium (DMEM) low glucose (Hyclone; Logan, UT) containing 10% Antibiotic/Antimycotic solution (AA, Gibco, USA) and 5% Amphotericin B (Gibco, USA) solution of 10 minutes each wash. The hypodermis was separated from the dermis, minced into small pieces, transferred to 50mL conical tubes with collagenase Type I (1mg/mL) and incubated for 1 hour at 37°C. The minced hypodermis was then filtered through a 70μm mesh filter. DMEM low glucose media (Hyclone; Logan, UT) with 10% fetal bovine serum (FBS) and 1% AA was added to the collected cell suspension, inactivating the enzyme. The tubes were centrifuged at 445.5 rcf for 5 minutes and the supernatant was discarded. The cells were seeded in tissue culture coated plates in a density of 8000 cells/cm^2^. The epidermis and dermis were incubated overnight at 37°C in 3–4 U/mL of Dispase II (Worthington Biochemical Corporation, US) in DMEM low glucose basal medium (Cytiva, US). After 12–14 hours, the epidermis was separated from the dermis. The epidermis tissue was finely minced, trypsinized, and poured through a mesh filter (70μm), followed by centrifugation at 198 rcf with half of the pellet resuspended in KGM2 medium for keratinocytes and half of the pellet resuspended in MelM medium for melanocytes (Gibco; Grand Island, NY). Splitting of the pellet and resuspension in separate media is due to melanocytes and keratinocytes favoring the respective selected medias for expansion. Similarly, the dermal tissue was minced, trypsinized, and poured through a nylon 70μm mesh, followed by centrifugation and resuspension in DMEM high glucose (HyClone; Logan, UT) with 10% FBS and 1% AA (Gibco, USA).

Endothelial cells (HUVECs, Cat.# C-12203) and Follicle dermal papilla cells (FDPCs, Cat.# C-12071) from pooled donors at passage 1 were purchased from PromoCell (Germany). All the cells were maintained as described below.

### Cell Culture

Keratinocytes were cultured in KGM-2 (Gibco; Grand Island, NY) and passaged at 70% confluence, and cells of passages 4–6 were used to make skin organoids. Melanocytes were cultured in MelM medium supplemented with SupplementMix (Sciencell, USA). Melanocytes were passaged at 90% confluence, and cells of passages 4–7 were used to make skin organoids.

The isolated fibroblasts were cultured in Dulbecco’s Modified Eagle Medium (DMEM) with high glucose (HyClone; Logan, UT) with 10% FBS (HyClone; Logan, UT) and 1% AA (Gibco; Grand Island, NY) supplements. Fibroblasts were passaged at 80% confluence and cells of passages 4–6 were used to generate SOs. HUVECs were cultured in Endothelial Growth Medium-2 supplemented with FBS, hydrocortisone, hFGF-b, VEGF, R3- IGF, ascorbic acid, hEGF, gentamycin, heparin (Lonza; Myersville, MD) and supplemented with 1% AA (Gibco; Grand Island, NY) solution. HUVECs were passaged at a confluence of 80%, with cells of passages 4–7 used to make SOs. FDPCs were cultured in Follicle Dermal Papilla Cell Growth Medium supplemented with Growth Medium SupplementMix (Promo Cell, USA). FDPCs were passaged at 90% confluence with cells from passages 4–8 used to make SOs.

For hypodermal cells, pre-adipocytes were cultured in Dulbecco’s Modified Eagle Medium (DMEM) with low glucose (HyClone; Logan, UT) supplemented 10% FBS (HyClone; Logan, UT) and 1% AA solution (Gibco; Grand Island, NY). Pre-adipocytes were passaged at 100% confluence with cells from passages 4–8 used to make SOs.

### Human skin organoid formation and maturation

All six cell types in 2D culture were washed twice with DPBS and lifted with 0.05% trypsin (Gibco, US) solution. The cell suspension was transferred to 50mL tubes and centrifuged 5min at 100g. The resulting pellets were resuspended in their corresponding growth medium. Cell counts were calculated using a hemocytometer. Skin organoids were made by mixing all six skin cell types together in specific ratios for each skin layer from Jorgensen et al (2023) for each skin layer – epidermis: human keratinocytes and human dark melanocytes (ratio, 9:1); dermis: human dermal fibroblast, human FDPCs, and HUVECs (ratio, 6:1:1); hypodermis: human pre-adipocytes (ratio, 1). Skin organoids were aggregated from in Ultra-Low Attachment 96 Well Plates (Corning Costar) by seeding 10,000 of mixed cells per well in a final volume of 150μl per well. The mixed culture medium containing a 1:1:1:1:1:1 ratio of each cell-specific culture medium was changed every 2 days.

### Brightfield imaging and SO area and volume calculating

Brightfield imaging of skin organoids was performed on days 7, 14, and 21 with an Olympus CKZ53 microscope using LC micro software (Japan). ImageJ software was used for surface area and volume calculations of 10 SOs at each time point. The diameter of the whole organoids was measured three times, and the average was applied to the formulas to calculate the overall organoid area and volume. To evaluate epidermal thickness, the diameter of the organoid core only (below the epidermal basal membrane) was measured three times, and its average was applied to the formula to calculate volume. The volume of the core only was subtracted from the volume of the overall organoid to assess differences in epidermal thickness.

### Live/dead viability assay

SO viability was analyzed on days 7, 14, and 21 of culture using LIVE/DEAD Viability/Cytotoxicity Kit (Invitrogen, City, State). Organoids were collected in centrifuge tubes and incubated with 2 μM Calcein AM and 4 μM Ethidium Homodimer solution for 1 hour at 37°C. The organoids were then transferred into Nunc^™^ Glass Bottom Dishes (ThermoFisher Scientific, USA), and analyzed using the confocal microscope Leica TCS LSI Macro (Leica, Germany).

### Scanning electron microscopy

The microstructure and morphology of the SOs was examined on day 7, 14 and 21 of culture. Organoids were fixed in glutaraldehyde (2.5% solution in DPBS, pH 7.4) overnight at 4°С. The samples were washed three times in DPBS, dehydrated in ethanol and dried using a critical point dryer (Leica EM CPD300) for approximately one hour. Skin organoids were then immobilized on a stub as whole samples or split in half and sputter-coated with particles of gold-palladium (Leica EM ACE600). The resulting replica was analyzed under a FlexSEM1000 scanning electron microscope (Hitachi).

### Histology

Sos were fixed in paraformaldehyde (4% solution in DPBS) overnight at 4°С, then embedded into agarose (3% solution in DPBS) and transferred to 70% ethanol prior to paraffin processing. A microtome (Leica) was used to generate 5 μm sections containing the full plane of the organoids. Slides were then stained with Hematoxylin (Sigma-Aldrich) and Eosin (Sigma-Aldrich), Masson’s Trichrome (Sigma-Aldrich), and Fontana-Masson (Sigma-Aldrich). Slides were imaged using brightfield microscopy. Images were then examined to determine the structure of the organoids, maturation of ECM and epidermal barrier formation.

### Immunohistochemistry (IHC)

Slides containing the sectioned organoids underwent antigen retrieval consisting of 10mM sodium citrate (pH = 6.0) for 2h. Individual tissue sections were delineated with a gel pen and permeabilized with PBS-Triton-X (0.2%) for 10min in a humidified chamber. After two 5-minute PBS washes, sections were incubated in protein-blocking solution (Dako; Carpinteria, CA) for 30min at room temperature. All primary antibodies (Table 1) were diluted in antibody diluent (Dako; Carpinteria, CA) 1 to 100 and incubated onto the sections overnight at 4°C. The following day, sections were washed three times in PBS and incubated with the secondary antibodies Alexa Fluor 488 anti-mouse (Abcam, cat. no. ab150117) and Alexa Fluor 594 anti-rabbit (Abcam, cat. no. ab150088) diluted 1 to 200 for 1h at room temperature. Following three more PBS washes, the slides were stained with 300nM DAPI and mounted with Prolong Gold without DAPI (Invitrogen; Eugene, OR). IHC slides were imaged at the fluorescence microscope Olympus BX63 using the CellSens Dimension software (Japan).

### Permeability assay

The integrity of the epithelial barrier was assessed as described previously with modifications (Rallabandi et al., 2020). The stock solution 1 mg/mL of FITC-dextran 4 kDa (Millipore Sigma, USA) was prepared by diluting the powder in Hank’s balanced salt solution (HBSS, Gibco, USA). 10 skin organoids per each test were collected in Nunc^™^ Glass Bottom Dishes (ThermoFisher Scientific, USA), washed twice with HBSS. Then, 25 ng/mL of FITC-dextran 4 kDa was added to each plate and the plates were incubated under normal growth conditions for 4 hours. As positive control we used 25 ng/mL of FITC-dextran 4 kDa was with 1% EDTA (ThermoFisher Scientific, USA). The permeability was observed using inner core absorption after 1- and 4-hours incubation under a confocal microscope Leica TCS LSI Macro (Leica, City, Germany), 5 samples per timepoint. The Integrated Fluorescent Intensity was quantified in ImageJ and compared between groups using statistical analysis software (GraphPad Prism, Graphpad Software Inc.).

### Retinol Metabolism

To test dermal bioavailability the SOs were treated with stabilized retinol similar to previously described *in vitro* models (Yourick et al., 2008; Li et al., 2017). 1 μM retinol (Millipore Sigma, US) was added to SOs’ medium on day 7, 14, and 21. Following 48h incubation, the media was collected for ELISA using the Human Retinoic ELISA kit (MyBiosource, USA). The untreated skin organoids were used as controls. Results were obtained from the spectrophotometer (SpectraMax M5, Molecular Devices; Sunnyvale, CA). A standard curve was made and used to determine the final Retinoic acid concentrations in each condition.

### Melanin Photometric Assay

Prior to photometric analysis, the samples of skin organoids (10 random organoids per time point) were washed three times from the residues of the medium in PBS (pH = 7.4), after which they were stored at − 20°C. To extract melanin, the samples were thawed and dried, and then 250 μl of SOLVABLE solution (6NE9100, PerkinElmer) was added to each sample to lysate them. Next, the samples were incubated for 18 h in a water bath at + 60°C. After incubation, the samples were thoroughly mixed in a vortex mixer, undissolved particles were precipitated by centrifugation (5 min, 13,000 g), 100 μl of the supernatant was placed into a 96-well plate, and samples’ optical densities were measured at a wavelength of 490 nm on a SpectraMax M5 Microplate Reader (Molecular Devices, USA). A calibration curve was obtained by analyzing standard solutions with a known concentration of eumelanin (M863, Sigma-Aldrich). Measurements were performed in triplicates.

### Chemical Irritation testing

Epidermal barrier resistance was tested through exposure to TritonX100 (Millipore Sigma, US). 7-day-old Sos were either incubated with DPBS (Cytiva, USA) (controls) or 1% TritonX100 (Millipore Sigma) for 6h. Samples were collected at 0.5h, 3h, and 6h of treatment and processed for histology and immunohistochemistry (10 organoids per time point and assay).

The rest of the chemical irritant treatments included a solution of 1% DMSO (Millipore Sigma, USA) diluted in DPBS, a 5% KOH solution (Sigma Aldrich; St. Louis, MO), Isopropanol (Sigma Aldrich; St. Louis, MO), and Hexyl Salicylate (Sigma Aldrich; St. Louis, MO). 7-day-old skin organoids were treated with one of the mentioned chemicals for 15min. After treatment, organoids were washed twice with DPBS for 5min. Then, fresh media was added. Organoid and media samples were collected at 24h and 48h after treatment and processed for live/dead analysis and ELISA, respectively. The Human IL-1alpha ELISA kit (Invitrogen, US) was used and results were obtained from the spectrophotometer (SpectraMax M5, Molecular Devices; Sunnyvale, CA). A standard curve was made and used to determine the final IL-1alpha concentrations in each condition.

### Ultraviolet B exposure

Control and experimental groups’ seven-day-old organoids were transferred in the phenol red-free media to prevent UVB absorption. The seven-day-old organoids were irradiated daily with a UVB lamp (Model KN-4006BL Philips UV Bulb) at a dose of 600mJ/cm^2^ for 4.5 minutes throughout 6 days. 24h after the last exposure, organoids were collected and compared to 14-day-old intact, non-exposed organoids. To generate the positive controls for ROS damage, intact non-UVB-exposed skin organoids were treated with 100μM Cobalt Chloride (Millipore Sigma, US) for 1h to induce ROS generation. To generate the UVB damage-rescued organoids, UVB-exposed organoids were treated with L-Ascorbic acid (Millipore Sigma, US) after each exposure, replacing the media with phenol red-free media containing ascorbic acid. For each group, a total of 96 organoids per condition were collected for further analysis, including live/dead staining, histology, immunochemistry, Comet assay, and rt-qPCR.

### Comet Assay or Single-Cell Gel Electrophoresis (SCGE assay)

The comet assay was performed to evaluate DNA damage in individual cells following the protocol outlined by Singh et al. (1988) with slight modifications. SOs were dissociated to single cells by incubating with Accutase (Gibco, USA) for 30 minutes at 37°C, then centrifuged at 198 rcf for 5 minutes. Cells were suspended in low-melting-point agarose (LMA, 0.5%, R&D Systems) and pipetted onto glass slides pre-coated with normal melting agarose (NMPA, 1%, ThermoFisher Scientific), then allowed to solidify. After solidification, a final third layer containing low melting agarose without cells was coated on top of the second layer, creating a sandwich structure. A glass coverslip was placed, and the slides were left to solidify for 20 minutes at 4°C. Following the removal of coverslips, slides underwent lysis in a pre-chilled buffer (1% sodium lauryl sarcosinate) for approximately 2 hours at 4°C. Post-lysis, the slides were incubated in electrophoresis buffer (10mM Tris, 1mM EDTA) for 30 minutes at 4°C and subjected to electrophoresis at a low voltage of 20V for 30 minutes set at 4°C. Subsequent steps included neutralization with a buffer (0.4M Tris) and dehydration. Comets were stained with ethidium bromide (20μg/mL) for visualization under a fluorescent microscope. For each condition, 100 randomly selected comets were used to quantify DNA damage using specialized software (CaspLab-, Comet assay software Project) (Końca et al., 2003), and tail length served as a measure of genotoxicity and cellular responses to treatments or exposures.

### Quantitative Real-Time PCR (qPCR) Analysis

For the qPCR analysis, organoids were collected in 1.5mL centrifuge tubes at different time points. After media removal, Trizol^®^ reagent (Thermo Fisher Scientific, Inc. USA) was added for lysis and standard protocols were meticulously followed for RNA extraction as per the manufacturer’s instructions. RNA quantification was carried out using the Nanodrop, and only samples exhibiting a 260/280 ratio greater than 2 were selected for cDNA synthesis. For samples with suboptimal RNA quality, the RNA cleanup kit from Qiagen was employed before proceeding with cDNA synthesis. Following cDNA synthesis using the High Capacity cDNA Reverse Transcription Kit (Applied Biosystems, USA), the samples underwent qPCR analysis utilizing Power SYBR^®^ Green PCR Master Mix (Applied Biosystems, USA) in Quantstudio 3 (Applied Biosystems, USA) by using the following PCR program: Stage 1: Activation: 50°C for 2 min; Stage 2: pre-soak:95°C for 10 min; Stage 3: Denaturation: 95°C for 15 sec, Annealing: 60°C for 1 min; Stage 4: Melting curve: 95°C for 15 sec, 60°C for 15 sec, 95°C for 15 sec. The qPCR primers (Table 2), essential for specific gene expression markers, were sourced from IDT (Integrated DNA Technologies, Inc. USA). The fold expression for the listed skin markers was calculated after normalizing to the housekeeping gene *GAPDH*. The analyzed genes covered various aspects of skin biology. Those associated with Epidermal Differentiation/Barrier Formation included *KRT10*, *FLG*, *CLDN1*, *CDSN*, and *CERS3*. Genes involved in Skin Development/Regulation comprised *SOX9*, *TGFB1*, and *TGFB3*. Furthermore, genes related to Extracellular Matrix and Tissue Remodeling encompassed *MMP3* and *TIMP1*. Additionally, *PPARG*, the gene associated with Skin Development/Adipogenesis, was examined. This comprehensive and systematic approach aimed to unravel the intricate processes occurring during the maturation of skin organoids, providing valuable insights into the dynamic gene expression patterns over the 21-day cultivation period.

### Statistics

All quantifiable data was expressed as the mean ± SD and statistical significance was determined using statistical analysis software (GraphPad Prism, Graphpad Software Inc.). For each assay ordinary one-way mixed models analysis of variance (ANOVA) with Tukey’s multiple comparisons were used to compare the group by time interaction effect. Statistical significance was defined as a p < 0.01.

## 3 Results

### Primary Skin Cells Self-organize Into Viable, Spherical, Layered Organoids

In this study, we employed an unconventional approach to develop a more intricate and physiologically accurate miniature *in vitro* skin model. In contrast to traditional skin constructs that build layer-by-layer, organoids rely on cell-cell crosstalk and self-organization to orchestrate its morphogenesis. To enrich the signal cue context in our model, we combined 6 different cell types, representative from all three layers of the skin (epidermis, dermis and hypodermis) under non-adhesive conditions in 96-well round-bottom plates, with a density of 10,000 cells per well in proportions, from Jorgensen, et al (2023), that mimic those of the native skin. Epidermal cells included keratinocytes and melanocytes. The dermal core contained fibroblasts, follicular dermal papilla cells (FDPCs) and endothelial cells whereas adipocytes composed the hypodermal component.

**Figure 1:**
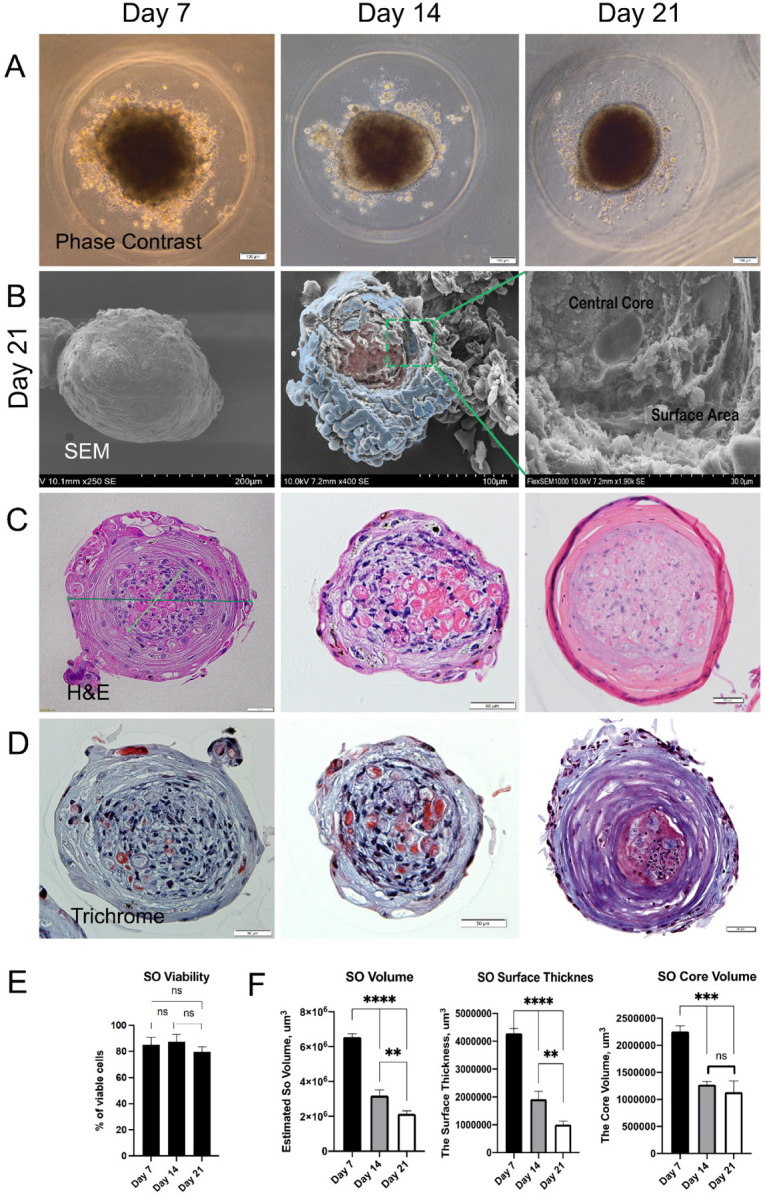
Primary skin cells self-organize into viable, spherical, layered organoids. A) Phase contrast microscopy of skin organoids in microwells over 21 days in culture. B) Scanning electron microscopy of organoids at 21 days of cultivation showed smooth surface morphology (left image) and distinct separation of central core (pink) and surface (blue) (center image). Enlarged section from central image showed compact, dense composition of central core and layered organization of surface area (right image). C) H&E staining confirmed layered structure in skin organoids at different time points. Example of diameter measurement in dark green, and core measurement in light green. D) Trichrome staining showed progressive, but nonsignificant, changes in collagen deposition within the organoids. E) Live/Dead cell staining determined cell viability as percent of live cells in skin organoids. F) Morphometric analysis of skin organoids’ volumes, surface thickness and core volumes over 21 days in culture. One-way ANOVA, **p<0,01, ***p<0,001, ****p<0,0001.

The aggregation of the distinct types of primary human skin cells led to their self-organization from loosely associated cells to compact spheres (Fig. 1A). The organoid surface underwent a progressive flattening and smoothening, as indicated by SEM images (Fig. 1B). Cells within the SOs showed high viability throughout the whole maturation process (Fig. 1E). Anatomically, the organoids were composed of two distinct zones that were preserved until day 21: an outer layer, resembling the epidermis, and an inner core (Fig. 1C). Trichrome staining showed an increase in collagen deposition from day 14 to day 21, indicative of dermal fibroblast activity (Fig. 1D). Throughout the culture period, we observed enucleated cells with keratinocyte morphological features in the organoid’s core. Morphometric analysis showed the progressive condensation of the organoids, reaching a significantly smaller size by day 21 (Fig. 1F). This size decrease was associated with a substantial and progressive reduction of the outer layer’s thickness, while the core’s volume reached its minimal size and stabilized by day 14. Together, these results indicate that the co-culture and aggregation conditions used here were conducive to the association and self-organization of the primary skin cells into layered spherical constructs.

### Formation of Epidermal and Dermal/Hypodermal Layers in Skin Organoids

**Figure 2:**
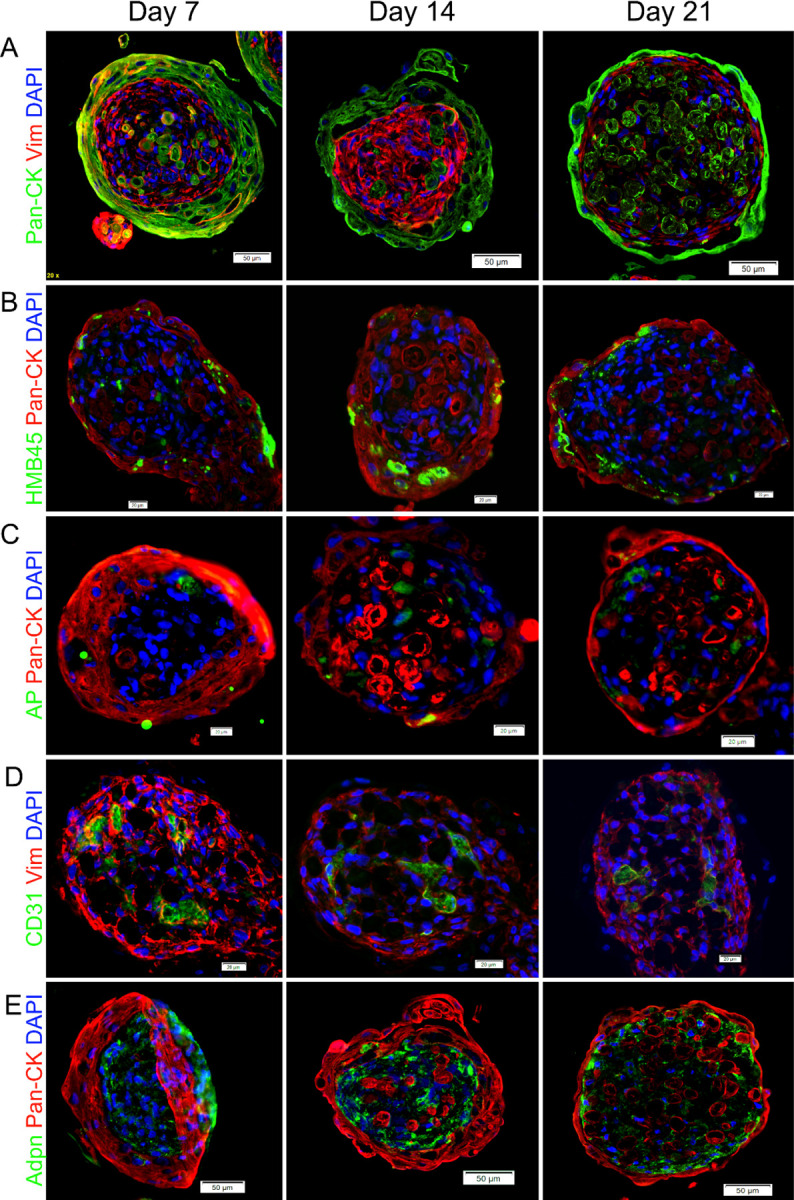
Spherical skin organoids recapitulate and maintain skin-like anatomy with an epidermal surface with keratinocytes surrounding a dermal/hypodermal core up to 21 days. A) Keratinocytes which formed the outer layer were marked by pan-cytokeratin (Pan-CK, green) and fibroblasts which comprised the dermal core were marked by vimentin (Vim, red). B) Melanocytes, marked by HBM45 (green), were distributed through the surface area among the keratinocytes stained by pan-cytokeratin (Pan-CK, red). C) FDPC was marked by alkaline phosphatase (AP, green) and shown to reside within the dermal core underneath the keratinocytes stained by pan-cytokeratin (Pan-CK, red). D) Endothelial cells marked by CD31 (green) formed clusters and tubule-like structures in the dermal core labelled by vimentin (Vim, red) during 21 days of cultivation. E) Hypodermal cells, adipocytes, labeled by adiponectin (green) staining were surrounded by keratinocytes stained by pan-cytokeratin (Pan-CK, red), which showed that the adipocytes were contained within the central core. Nuclei were counterstained by DAPI (blue) in all images. *Immunohistochemistry, fluorescent microscopy*.

To verify the development of the skin layers in our organoids, we conducted immunochemistry (IHC) analysis against cell type-specific markers (Fig. 2).

The outer layers of the SOs consisted primarily of epidermal keratinocytes, stained with Pan-cytokeratin (Pan-CK), and HMB45 + melanocytes. In the central core, the majority of the cells were stained with vimentin (Vim) and adiponectin, which are markers of dermal and hypodermal cells, such as fibroblasts and adipocytes, respectively. Alkaline phosphatase (AP)-expressing FDPCs and CD31 + endothelial cells were also observed in the central zone. Enucleated cells detected by histological staining in the core were identified as keratinocytes. This skin-like cell arrangement was maintained over 21 days of SO cultivation (Fig. 2). However, some morphological changes were observed between day 7 and day 21. As the epidermal layer matured, it showed progressive thinning and appearance of enucleated keratinocytes and melanosomes over time. The most prominent morphological change in the derma-hypodermal core was the aggregation of endothelial cells into spherical clusters, which began to form tubular like structures, indicating the beginning of vasculogenesis.

### Development of Basement Membrane and Establishing of Stratified Epidermis in Skin Organoids

**Figure 3:**
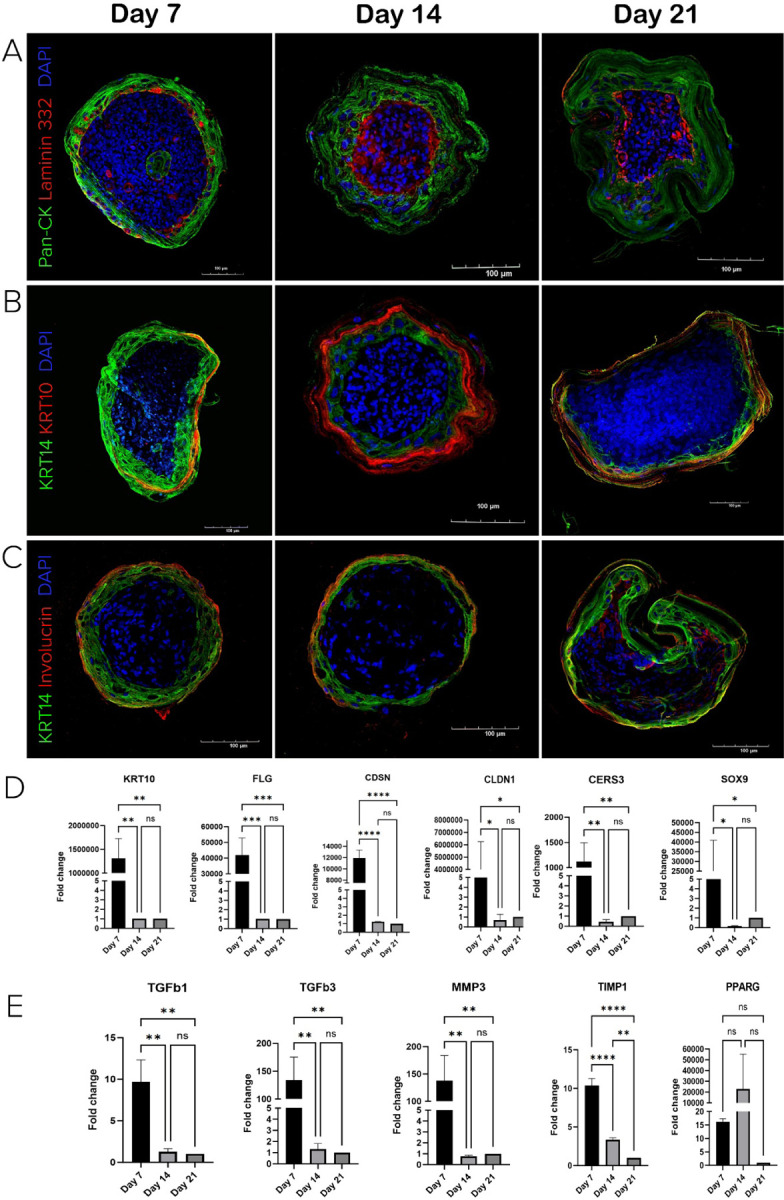
Skin Organoids show basement membrane development and stratification in surface area. A) SOs showed progressive development of basement membrane stained by Laminin 332 (red) underneath keratinocytes marked by pan-cytokeratin (green). B) Surface area showed stratification with basal cells marked by Keratin 14 (KRT14, green) and differentiated cells labeled by Keratin 10 (KRT10, red) C) SO displayed cell differentiation with Involucrin positive cells (red) and basal cells stained by Keratin 14 (KRT14, green). D) Gene expression of epidermal maturation markers in skin organoids. *qPCR analysis*. E) Gene expression of ECM remodeling factors and adipocyte differentiation marker PPARG in skin organoids over 21 days in culture. *qPCR analysis*. One-way ANOVA, **p<0,01, ***p<0,001, ****p<0,0001.

The surface layer of the skin organoids showed the formation of typical epidermal features including the development of a basement membrane and stratification within the outer layer. SOs showed accumulation of laminin 332 protein (red) between the core and the surface area over 21 days (Fig. 3A). Keratinocytes residing on top of the basement membrane were confirmed as basal progenitor cells by Keratin 14 marker (Fig. 3B, C, green). Keratin 10 staining revealed the presence of differentiated keratinocytes in the outermost layers of the organoids on day 7 that grew more distinct over time (Fig. 3B, red). The maturation of epidermal layer and beginning of cornification was indicated by involucrin expression that appeared on day 7 and progressed over 21 days (Fig. 3C, red).

We next examined the dynamic behavior of specific genes associated with various aspects of skin biology, including epidermal differentiation, barrier formation, skin development, regulation, extracellular matrix dynamics, tissue remodeling and adipogenesis throughout the 21-day culture period. We compared gene expression levels on days 7 and 14 to day 21, as the latter represented the most mature state of the SOs (Fig. 3D, E). Gene expression analysis of epidermal development revealed significantly higher expression levels of markers for epidermis stratification (*KRT10, FLG, CDSN*), epidermal barrier development (*CLDN1, CERS3*), and melanocyte differentiation (*SOX9*) on day 7 compared to day 14 and day 21 (Fig. 3D). A similar progressive reduction in expression was shown for the ECM remodeling factors such as collagen secretion and remodeling growth factors (*TGFb1, TGFb3*), enzymes such as metalloproteinase 3 (*MMP3*), and tissue inhibitor of metalloproteinase (*TIMP1*) with a peak of gene expression on day 7 and decreasing levels on day 14 and day 21 (Fig. 3E). The adipocyte differentiation marker *PPARG* showed a non-statistically significant increase in expression on day 14. Together, these results confirm our prior observation of progressive maturation of the skin organoids over 21 days and suggest that the key intrinsic molecular events are initiated during the first week of skin organoid maturation.

### Skin Organoids Accurately Replicate the Physiological Functions of Native Skin.

To determine if the observed morphological changes are accompanied with the functional development of skin organoids, we assessed various physiological skin properties in the organoids such as barrier function, pigmentation, vascularization, and retinol metabolism.

#### 3.1.1 Epidermal Barrier Formation in Skin Organoids

The main function of the skin is to serve as a barrier against the exterior. Thus, its integrity is crucial for maintaining skin health protecting the body from dehydration and external threats such as pathogens, allergens, and environmental toxins. Impairment of the skin barrier results in a range of skin disorders such as eczema, psoriasis, and dermatitis. Studying the impact of different treatments on the skin barrier *in vitro* can reveal and reduce the usage of irritating and cancerogenic chemicals. This approach can also facilitate the development of precise therapies that restore the integrity and functionality of the skin barrier, leading to better outcomes for patients with skin diseases (Baker et al., 2023).

**Figure 4:**
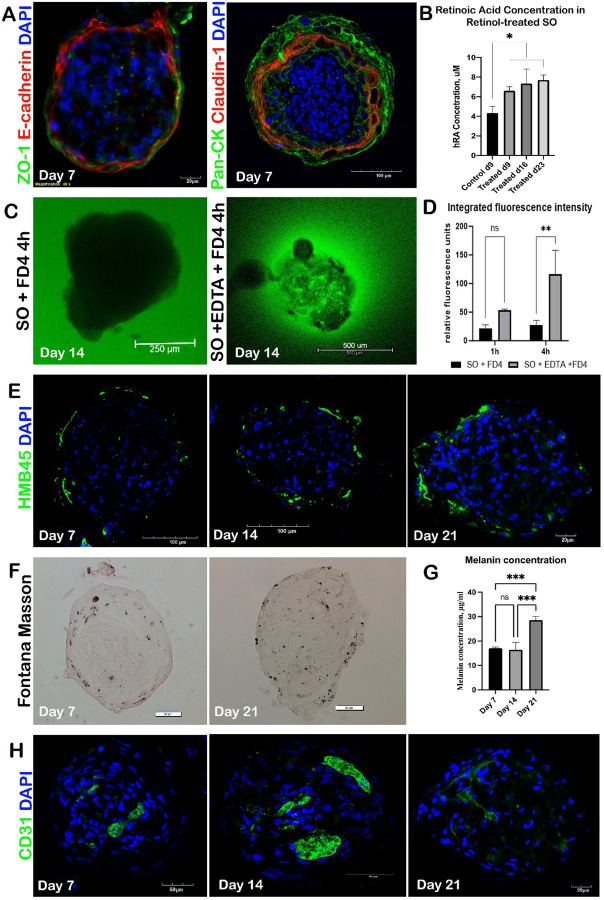
Skin organoids display key morpho-functional features, including an epidermal barrier, pigmentation, organization of CD31^+^ endothelial cells, and retinol metabolism. A) On day 7, SOs showed formation of tight (ZO-1, green; Claudin-1, red) and adherens (E-cadherin, red) junctions between keratinocytes stained by pan-cytokeratin (Pan-CK, green). Nuclei are counterstained by DAPI (blue). B) SOs exhibited the ability to metabolize retinol as shown by ELISA assays of retinoic acid concentration in skin organoids over 21 days of 3D cultivation. One-way ANOVA, *p<0,01. C) On day 14, SOs displayed robust epidermal barrier function in intact SOs compared to EDTA-treated SOs as shown by incubation with 4 kDa Dextran-FITC (FD4, green) for 4 hours. D) EDTA-treated SOs showed significantly increased fluorescent intensity in central area conversely to untreated SOs. One-way ANOVA, **p<0,001 E) SOs demonstrated a stable synthesis of early-stage melanosomes marked by HBM45 (green). Nuclei are counterstained by DAPI (blue). F) Fontana-Masson staining revealed accumulation of eumelanin mostly in the surface area of skin organoids during cultivation. G) Increasing melanin concentration in skin organoids during cultivation from day 7 to day 21 was confirmed by Photometric assay. One-way ANOVA, *p<0,01, ***p<0,0001, ****p<0,00001. H) SOs showed the organization of CD31-positive (green) endothelial cells from the formation of spherical clusters, by day 7, to cord-like structures in the central core, by day 14 to day 21, which may be initial indications of vasculogenesis.

Co-staining for E-cadherin, ZO-1 and Claudin − 1 demonstrated the development of inter-keratinocyte adherens and tight junctions which provide the barrier function in the native skin (Fig. 4A). The SOs showed an effective barrier function by preventing the penetration of fluorescently labeled 4kDa Dextran-FITC into the organoid core after 4h of incubation. In contrast, in EDTA-treated organoids, the epidermis became permeable, as evidenced by the significant accumulation of the fluorescent dye observed inside the organoids (Fig. 4C, D). Overall, the SOs formed an outer layer with both anatomical and functional epidermal properties.

#### 3.1.2 Pigmentation in Skin organoids

Keratinocytes play a crucial role in the mechanical protection of skin, whereas melanocytes are vital in creating a shield against UV radiation. UV radiation is highly detrimental and can lead to sunburns, photodamage, chronic inflammation, formation of reactive oxygen species (ROS), and cancer development. Melanocytes produce and distribute the pigment melanin, which hinders the entry of UV light into the innermost layers of the skin (Brenner and Hearing, 2008). Examining pigmentation in skin models in a laboratory setting can facilitate the identification of novel molecular/cellular events associated with UV-induced damage, as well as the development of innovative approaches for managing hypo- and hyperpigmentation disorders.

To assess the production and accumulation of melanin in skin organoids, we used HMB45 to label *de novo* generated melanosomes. We observed a progressive increase of the signal at the periphery of the maturing organoids (Fig. 4E). Fontana-Masson staining demonstrated the production of the dark pigment eumelanin, and its distribution mostly in the outer layers of the skin organoid (Fig. 4F). Melanin concentration increased over time during the organoids’ culture and peaked at day 21 as revealed by the Photometric assay (Fig. 4G). These findings indicate the SOs’ ability to develop pigmentation *de novo*.

#### 3.1.3 Endothelial Cell Assembly in Skin Organoids

In the in vitro skin models, endothelial cells have the potential to create vascularization whose importance lies in its ability to mimic the physiological role of microvessels (Rimal et al., 2024). Incorporation of a vascular network within these models, would allow researchers to better understand how blood vessels interact with regenerating tissues and assess the efficacy of potential therapies aimed at enhancing vascularization. This knowledge can ultimately contribute to developing more accurate and effective in vitro models for studying skin regeneration and aid in discovering novel treatments for various skin conditions or injuries.

Between days 7 and 21 of culture, we observed changes in the behavior pattern of CD31 + endothelial cells that gave indications of the initial stage of vasculogenesis may have begun. On day 7, CD31 + endothelial cells were aggregated into spherical clusters at the center organoid. Further, from day 14 to day 21 they formed sprouts which migrated through the organoid’s core to form elongated cord-like structures (Fig. 4H).

#### 3.1.4 Retinol Metabolism in Skin Organoids

We assessed skin function to absorb and metabolize active ingredients by introducing retinol to the skin organoid system. Retinol metabolism plays a crucial role in maintaining the overall health and function of the skin (Zasada et al., 2019). It involves various physiological processes, such as cell growth, differentiation, and collagen production. Additionally, retinol metabolism is closely linked to the development and progression of skin diseases, including acne, psoriasis, and photoaging. Therefore, in vitro skin models that replicate retinol utilization can lead to an understanding of the intricate mechanisms of retinol metabolism and can pave the way for targeted therapeutic interventions to address skin-related issues and promote optimal skin health.

Retinol metabolism was assessed by introducing retinol into the culture medium of organoids at different stages of development (days in culture). We observed a substantial elevation in retinoic acid levels in the skin organoids 48 hours post-treatment measured at all development stages, compared with controls (Fig. 4B), which demonstrated the organoids’ capacity to metabolize retinol akin to the *in vivo* skin.

### Modeling Chemical Irritation in Skin Organoids

The need for an in vitro skin irritation model with human cells is crucial in the field of dermatology and cosmetic industries. Such models allow us to study and understand the effects of various substances on the skin more accurately than animal models, helping in the development of safer and more effective skincare products (Portugal-Cohen et al., 2023). Additionally, a reliable skin irritation model can also aid in the identification and evaluation of potential allergens, providing valuable insights for regulatory purposes and consumer safety.

**Figure 5:**
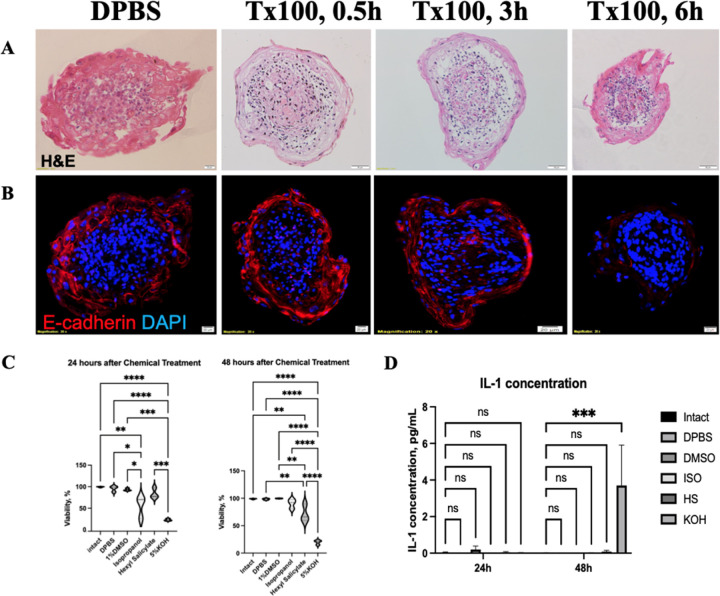
Skin Organoid resistance to irritants. A, B) H&E (A) and E-cadherin (B, red) staining of skin organoids exposed to 1% Triton, at different times post exposure, showing separation of the outer layer from the core after 3 hours exposure. C) Cell viability inside skin organoids exposed to irritant chemicals, as determine by Live/Dead staining, 24h and 48h post exposure one-way ANOVA, *p<0.05, **p<0.01, ***p<0.001. D) IL-1 secretion form – Skin organoid, as measure by ELISA. 24h and 48h post exposure one-way ANOVA, ***p<0.001.

To assess the feasibility of using SOs as a model for chemical irritation we first tested the resistance of the epidermal barrier. Triton was selected for initial testing following OECD recommendations for selection of chemicals for initial testing (OECD, 2021). We induced skin barrier destruction by incubating 7-day old SOs in a 1% Triton solution for increasing periods of time. H&E staining demonstrated that the organoids remained unchanged at 0.5 hours, exhibited only partial detachment of the outer layer at 3 hours, and complete disappearance of the outer layer at 6 hours incubation (Fig. 5A). E-cadherin immunostaining showed continuity of cell junctions in the epidermal layer confirming its integrity at 3 hours and a progressive disruption of the intercellular connections in the outer layers through 6 hours of incubation (Fig. 5B). These results suggest that the organoid epidermal barrier has a strong resistance to the Triton solution.

Next, we examined the effects of other skin irritants, following the OECD recommendations for *in vitro* skin models (OECD, 2021). To determine the irritating effects of Isopropanol, Hexyl Salicylate, and 5% KOH we exposed skin organoids to these compounds for 15min and analyzed cell viability and IL-1 secretion 24 and 48h post-exposure. As per the OECD rules, a chemical is classified as highly irritating if it induced > 50% reduction in cell viability within 48 hours of treatment. DPBS and 1%DMSO solutions were employed as control measures and exhibited no notable disparity when compared to the nonexposed intact group. At 24-hours following chemical treatment, the skin organoids showed cell viability of 55.5% for the isopropanol group and 83.9% for the Hexyl Salicylate group (Fig. 5C), both higher than 50% whereas the 5% KOH group had a viability of only 22.83% (Fig. 5C). At 48 hours following post-treatment, viability increased for Isopropanol (90.16%) and r Hexyl Salicylate (71.7%), and slightly decreased for 5% KOH (20.0%) (Fig. 5C). The levels of secreted IL-1, a known marker of skin inflammation, were low and not significantly different than controls for all irritants after 24 hours but were robustly and significantly higher in 5% KOH-treated organoids after 48 hours, indicating a high irritancy (Fig. 5D). Therefore, these results are in line with each compound’s effect on *in vivo* skin, emphasizing the potential of these organoids as a chemical testing tool.

### Skin Organoid as a model of UVB-induced Injury

Sun activity has led to an increase in UV skin exposure, underscoring the importance of generating relevant models to investigate harmful effects and develop strategies to protect skin from damage (Marionnet et al., 2017). Advanced UV models would enable researchers to investigate the precise mechanisms by which UV radiation impacts the skin, including DNA damage and oxidative stress. These models also help assess the effectiveness of sunscreen formulations or other preventative measures. Moreover, a dependable UV exposure model may be of benefit to public health initiatives by allowing investigation of crucial information regarding the hazards linked to excessive sun exposure and directing suggestions for sun protection.

To test if our SOs develop anatomical and physiological responses to UVB exposure, we subjected 7-day-old skin organoids to daily low doses (600mJ/cm^2^, 4.5 min) of UVB radiation for a period of 7 days. Since UVB is expected to generate oxidative stress in exposed tissues, we generated positive controls by inducing oxidative stress through exposure of 14-day-old skin organoids to Cobalt Chloride for 1 hour. Intact SOs were utilized as a negative control group. To simulate reversal of low-dose UVB-induced damage, we treated a set of organoids with the antioxidant Ascorbic acid after every exposure to UVB.

**Figure 6:**
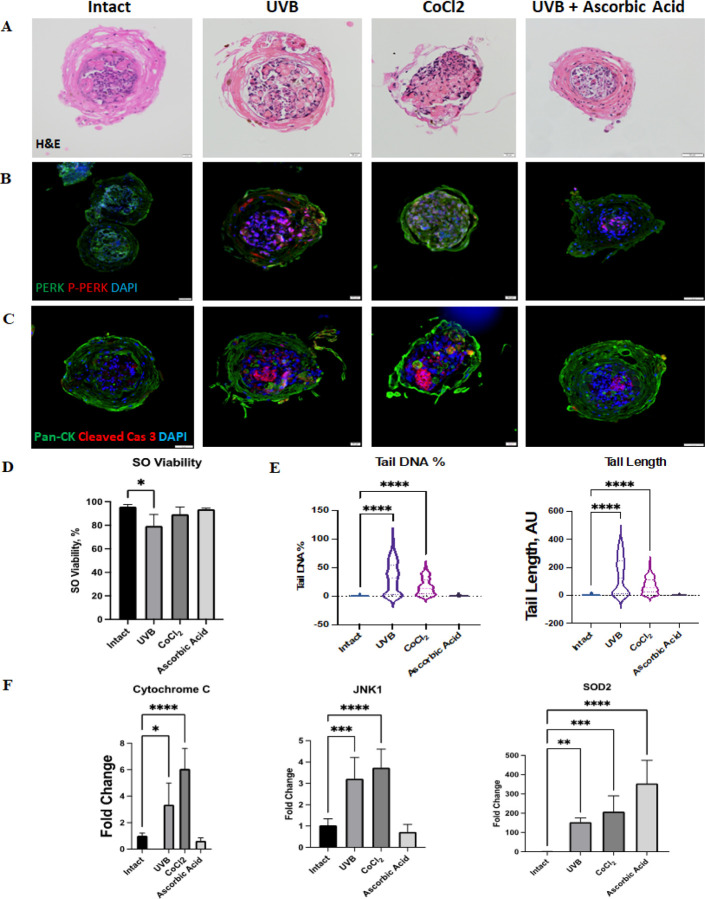
Pathological response of skin organoid to UVB exposure and oxidative stress. A-C) Anatomical changes in skin organoid morphology in response to UVB, Cobalt Chloride and ascorbic acid exposure. A - H&E staining, B – PERK (green) and P-PERK (red) staining, C - Cleaved caspase 3 (red) staining. D) Cell viability in organoids exposed to UVB, Cobalt Chloride and ascorbic acid, as determined by Live/Dead assay. (one-way ANOVA, *p<0.05, **p<0.01, ***p<0.001). E) DNA damage in skin organoids exposed to UVB, CoCl_2_ and ascorbic acid, as determined by Comet assay. (one-way ANOVA, *p<0.05, **p<0.01, ***p<0.001). F) Gene expression analysis of ER-stress marker *Cytochrome C*, apoptosis markers – *JNK*, and oxidative stress markers – *SOD2* in skin organoids exposed to UVB, Cobalt Chloride and ascorbic acid. (one-way ANOVA, *p<0.05, **p<0.01, ***p<0.001).

H&E staining of organoids after 6 days of daily administered low doses of UVB did not reveal significant anatomical change (Fig. 6A). However, Cobalt Chloride induced the breakdown of the outer layer of the SOs, while ascorbic acid maintained the thickness and stratification of the outer epidermal layer. These results suggest that our SOs anatomically respond to oxidative stress and antioxidant treatments. Pan-Cytokeratin IHC staining confirmed the histological observations, as the Cobalt Chloride group exhibited a reduced number of keratinocytes, while the Ascorbic Acid group had an increase in the thickness of the epidermal layer (Fig. 5B, C). To better understand the cellular process involved in these anatomical changes, we performed IHC staining for phosphorylated PERK (P-PERK, red) - the active version of PERK (green) as an indication of developing ER stress. The results showed higher P-PERK expression in the central zone of organoids exposed to UVB without ascorbic acid treatment, and Cobalt Chloride groups, consistent with the cellular effects of these two agents (Fig. 6B). Further cellular damage in these groups was confirmed by the staining for the apoptosis marker cleaved caspase 3, which revealed the presence of a highly apoptotic core in skin organoids exposed to UVB radiation and Cobalt Chloride, compared to controls (Fig. 6C). Live/Dead assay revealed approximately 20% reduction in cell viability only in UVB-irradiated organoids (Fig. 6D) Conversely, Ascorbic acid was able to prevent ER-stress and apoptosis, emulating its antioxidant effects in *in vivo* skin. qPCR confirmed these observations regarding ER stress, apoptosis, and oxidative stress in the UVB and Cobalt Chloride treated organoids through a statistically significant increase in the expression of *Cytochrome C*, *JNK1*, and *SOD2*, respectively (Fig. 5F). Ascorbic acid, on the other hand, induced the expression of superoxide dismutase 2 enzyme *SOD2*, as expected from an antioxidant agent.

Since UV-exposure can cause DNA damage and mutations, we used the comet assay to measure the amount of DNA damage in the skin organoids. UVB and Cobalt Chloride exposure induced significantly higher tail DNA percentage and tail length in cell nuclei in the skin organoids, indicative of severe DNA damage (Fig. 6E). Conversely, Ascorbic acid treatment showed no evidence of DNA damage. Together these results demonstrate the ability of our organoids to recapitulate the pathological consequences of native skin to UVB exposure and oxidative stress.

## 4 Discussion

Most available 3D skin models are fabricated layer-by-layer and then subjected to an air-liquid interface to stimulate epidermal differentiation (Footner et al, 2023). These models are labor-intensive and are restricted to a limited number of skin cell types, limiting the reproducibility of the cellular and structural complexity of human skin. To overcome these limitations, we previously utilized a bioprinting process for simultaneous *ex vivo* fabrication of full-thickness skin equivalents with six different cell types and showed accelerated healing in full-excision wounds (Jorgensen et al., 2020; Jorgensen et al., 2023). Building on our earlier success, in this study, we developed a newly designed skin organoid system, made of six primary skin cell types, that facilitated self- and cross-inductive cellular interactions to drive tissue-specific morphogenesis, reproducing skin-like microanatomy and functionality.

Previously described SO models showed epithelial cells in the innermost layer, encircled by dermal cells (Lei et al., 2017; Lee et al., 2018; Su et al., 2019; Diao et al., 2019). A similar cell distribution was also shown for intestine (Hou et al., 2018) and mammary (Sumbal et al., 2020) organoids and was considered typical for organoids containing epithelial cells. This organization aligns with the ‘differential adhesion’ hypothesis (DAH) (Fagotto, 2014; Barresi and Gilbert, 2020), that suggests that cell populations establish tissue-like boundaries based on surface tension differences in order to achieve minimal energy of interfacial interaction in 3D systems. In contrast, our cell aggregation approach showed autonomous assembly primary postnatal human skin cells into organized spheres with two distinct microanatomical regions. Due to the cell’s selective affinity (Barresi and Gilbert, 2020; Steinberg et al., 2004), the organoid system generates a structure that resembles the native skin, with an outer layer consisting of epidermal cells, and an inner core composed of dermal and hypodermal cells, highlighting the importance of cell diversity to decrease the difference in surface tension and develop a skin-like microanatomy. Due to the absence of the air-liquid interface, epidermal differentiation differs from conventional *in vitro* skin models, however, it retains robust features of mechanical integrity, pigmentation, and absorption. The use of alternative signal cues, such as ascorbic acid, can increase epidermal thickness and stratification.

Based on the gene expression, histology, and immunostaining results from our study, the epidermis and dermis morphogenesis process is completed by day 7, when keratinocytes obtained apical-basal polarity as indicated by the establishment of inter-cellular tight junctions and the formation of a basement membrane (Muroyama et al., 2012). The expression Keratin 10 in the outmost layers shows the skin-like differentiation and stratification in the surface area (Footner et al., 2023). Additionally, the presence of involucrin in the outermost keratinocytes on day 7 indicates the development of the cornified envelope by the same time (Candi et al., 2005). The generated SOs exhibited epidermal morphological maturation through continuous condensation and cell flattening in the surface area over 21 days. This deviates from the usual process of epidermal maturation seen in postnatal skin and *in vitro* skin models (Footner et al., 2023), which is typically accomplished through the extensive proliferation and delamination of keratinocytes (Damen et al., 2021). Epidermal changes observed in our system bear similarity to the initial cornification process during embryogenesis facilitated by periderm cells. During development, the embryonic ectoderm consists of a single squamous epithelium covered by flat and polygonal periderm cells that produce involucrin and prevent pathological epithelial attachment (Richardson et al., 2014). Like our model, as the ectoderm continues to mature, the periderm cells gradually retreat and decrease in number (Akiyama et al., 1999). Furthermore, the epidermal barrier of the SOs effectively prevented Dextran-FITC penetration, indicating the mechanical integrity of the surface layer akin to other well-established *in vitro* skin models (Roger et al., 2019; Footner et al., 2023). In contrast, through exposure to EDTA we significantly enhanced the barrier’s permeability, simulating various skin illnesses that demonstrate a compromised epidermal barrier. Overall, the SOs exhibited both anatomical and functional epidermal properties. Another example of the proper physiology of the SO is the inclusion of ascorbic acid in UVB exposure models, where epidermal maturation and stratification is induced, consistent with *in vivo* observations (Gref et al., 2020). Although conventional assays for skin absorption properties through diffusion do not apply to spherical models (OECD, 2004), we could demonstrate an alternative dermal bioavailability assessment method. We successfully showed retinol (vitamin A) absorption and retinoic acid metabolism which resemble normal skin, similar to previously used *in vitro* and *ex vivo* skin models (Zasada et al., 2019; Yourick et al., 2008; Li et al., 2017).

One of the major findings of the current study is pigmentation in the skin organoids. Our data suggests that melanin production and accumulation occur predominantly in the outer layers of the skin organoids, thus indicating their ability to develop pigmentation that is comparable to both natural skin and previously created skin models (Gledhill t al., 2015; Branquinho et al., 2020; Zoio et al., 2021; Goncalves et al., 2022; Cohen et al., 2023; Goncalves et al., 2023). The increase in melanin concentration in conjunction with the positive staining for HMB45 (PMEL17), a marker for stage I and II melanosomes in fetal and activated postnatal progenitor melanocytes but absent in the adult resting melanocytes (Kikuchi et al., 1996; Michalak-Mićka et al., 2022), indicates that our system undergoes active melanogenesis in an embryo like manner, which is consistent with using neonatal melanocytes. Moreover, our novel SO design appears to have generated a favorable microenvironment for the embryo-like development of microvessels in the organoid core. This process, known as vasculogenesis, begins with the formation of spherical clusters of endothelial cells and eventually leads to the establishment of a primitive tubule-like network via sprouting (Chung, Ferrara, 2011). Follow on experiments whether the tubule-like structures were blood vessels, or only mimicked blood vessels, in the early stages of vasculogenesis is necessary, including whether there was the formation of lumen in the structures. Overall, this study shows that the newly generated SOs exhibit both microanatomical and physiological features typical for embryonic skin development.

We also explored the feasibility of SOs for modeling external skin damage using chemical and UVB radiation insults. Following the standard guidelines for skin chemical irritancy tests *in vitro* (OECD, 2021), SOs demonstrate a strong resistance to Triton, a very toxic detergent, suggesting a durable epidermal structure similar to other skin models that resist incubation for up to 4 hours (Hoffmann et al., 2005; do Nascimento Pedrosa et al., 2017). These findings indicate that SOs can withstand rapid penetration of chemical compounds. Test results of chemical compounds with varying irritancy levels (OECD, 2021), aligned with the specified primary indexes and previous published studies (Jírová et al., 2010; Morales et al., 2016; Mewes et al., 2016; Portugal-Cohen et al., 2023). Moreover, SOs responded to the irritating KOH solution by releasing the pro-inflammatory cytokine IL-1 alpha into the media, similar to other hazardous chemicals in earlier studies (Walters et al., 2016; Olsen et al., 2018; Pellevoisin et al., 2018). Thus, the SO system shows potential use in skin irritation testing.

The minimal erythema dose (MED), the minimal dose which will cause a visible skin erythema, varies based on skin type and ranges from 13 to 156 mJ/cm^2^ (Dornelles et al., 2004), with average annual UV doses for indoor workers between 12.8 and 230.7 in the United States (Godar, 2004). Challenging our SOs with repetitive application of UVB radiation equivalent to between 3.8 and 46.2 MEDs daily, showed no morphological changes in skin microanatomy, but demonstrate characteristic of DNA damage, oxidative stress, and apoptosis, consistent with studies for both normal skin (Lin et al., 2019; Tanveer et al., 2023) and other *in vitro* skin models (Bernerd et al., 2008; Portugal-Cohen et al., 2011; Marionnet et al., 2015; Karapetsas et al., 2019). The UVB-induced pathological changes were similar to those seen in SOs treated with ROS-inducer Cobalt Chloride (He et al., 2018), suggesting that the main damage occurs via ROS generation. Furthermore, this was verified when ascorbic acid that functions as an electron donor and inhibits the generation of ROS (Kawashima et al., 2018) eliminated DNA damage and reduced oxidative stress, and apoptosis in SOs. Notably, we established that UVB radiation induces Endoplasmic Reticulum (ER) stress via phosphorylation of protein kinase RNA-like ER kinase (PERK) in our organoids, which has not been described before in 3D models. Our findings confirm previous results determined in cell monolayers in which UVB radiation induces the phosphorylation of α subunit of eukaryotic initiation factor 2 (elF2α) by activating PERK (Lu et al., 2009; Lin et al., 2019; Lorca et al., 2021). In summary, our novel UVB damage model accurately represents common processes caused by UVB radiation and enables the discovery of new mechanisms of photodamage and skin regeneration after it.

## Conclusion

Our study demonstrates that these novel SOs, composed of the 6 main cell types present in skin, have both microanatomical and physiological characteristics associated with skin, including the native skin cell type distribution. This model condenses a few weeks of human skin development into a few days. Skin-like self-assembly is a promising tool for studying skin development, physiology, and homeostasis changes triggered by diverse substances that enables discovery of new skin regeneration and disease pathways. The described SO model is suitable for chemical agent testing, providing valuable insights for producing safer and more effective products or may aid in toxicology studies. In conclusion, SOs, as an immersive model, are more suited for incorporation into the body-on-a-chip system and allow for speedier, high-throughput testing *in situ*. This unique model holds promise to study skin regeneration and disease modeling.

## Figures and Tables

**Table 1:** List of the antibodies used in the study.

Primary Antibody	Target Cell	Reference	Dilution
Pan-cytokeratin	Keratinocytes	Invitrogen cat. no. PA1–27114	1:100
Pan-cytokeratin	Keratinocytes	Thermo Fisher Scientific cat. no. RBMP49–7249-P1	1:100
Keratin 10 (KRT10)	Keratinocytes	Abcam cat. no. ab76318	1:100
Keratin 14 (KRT14)	Keratinocytes	Thermo Fisher Scientific cat. no. MA5–11599	1:100
HMB45	Melanocytes	Dako cat. no. M0634	1:100
Adiponectin	Adipocytes	Invitrogen cat. No. MA1–054	1:100
Vimentin	Fibroblasts	Invitrogen cat. no. MA5–16409	1:100
Alkaline Phosphatase	Follicle Dermal Papilla Cells (FDPCs)	Invitrogen cat. no. PA5–22210	1:50
Claudin-1	Tight Junctions	Abcam cat. no. ab211737	1:100
E-cadherin	Adherens Junctions	Abcam cat no. ab0772	1:100
ZO-1	Tight Junctions	Invitrogen cat. no. 33–9100	1:50
PERK	Endoplasmic reticulum	Invitrogen cat. no. MA5–15705	1:100
P-PERK	Endoplasmic reticulum stress	Invitrogen cat. no. PA5–40294	1:100
Cleaved Caspase 3	Apoptosis	Cell Signaling Technology cat. no. 9661	1:100
Involucrin	Differentiated Keratinocytes	Thermo Fisher Scientific cat. no. PA5–32454	1:100
Laminin 332	Skin Basement Membrane	Invitrogen cat. no. 711306	1:100
CD31	Endothelial Cells	NovusBio cat. no. NBP2–15188	1:50

**Table 2:** List of the primers used in the study, including primer sequences and amplicon sizes.

Gene	Biological Process	Forward Primer (‘5→3’)	Reverse Primer (‘5→3’)	Size (bp)
KRT10	Epidermal Differentiation/Barrier Formation	ACTACTCTTCCTCCCGCAGT	CAGAGCTCCCACGGCTAAAA	159
*FLG*		AGACACTCTCAGCACGGAAG	TGGCCACATAAACCTGGGTC	149
*CLDN1*		ATGACCCCAGTCAATGCCAG	GGCCTTGGTGTTGGGTAAGA	140
*CDSN*		TCTGGTCACCCTTGCATGTC	GAGGCTTCACTTGGGCTAGG	163
*CERS3*		CTCGCACAGATGGTGTCCTG	GCTTCCTGTTTCACCACTTGT	136
*SOX9*	Skin Development/Regulation	GGGCAAGCTCTGGAGACTTCTG	CTGCCCGTTCTTCACCGACT	142
*TGFB1*		CGACTCGCCAGAGTGGTTAT	CCGGTAGTGAACCCGTTGAT	156
*TGFB3*		GGCTGTCTGCCCTAAAGGAA	CTGGCCGAAGGATCTGGAAG	170
*MMP3*	Extracellular Matrix and Tissue Remodeling	ACAAAGGATACAACAGGGACCAA	ACCGAGTCAGGTCTGTGAGT	136
*TIMP1*		ATTCCGACCTCGTCATCAGG	GCATCCCCTAAGGCTTGGAA	124
*PPARG*	Skin Development/Adipogenesis	AGCAAACCCCTATTCCATGCT	TGTGTCAACCATGGTCATTTCTTG	126
*GAPDH*	Housekeeping Gene	GCTCTCTGCTCCTCCTGTTC	GCCCAATACGACCAAATCCGT	
